# Systems-based approach to examine the cytokine responses in primary mouse lung macrophages infected with low pathogenic avian Influenza virus circulating in South East Asia

**DOI:** 10.1186/s12864-017-3803-6

**Published:** 2017-05-30

**Authors:** Biruhalem Taye, Hui Chen, Myint Zu Myaing, Boon Huan Tan, Sebastian Maurer-Stroh, Richard J. Sugrue

**Affiliations:** 1 Bioinformatics Institute, A*STAR, 30 Biopolis Street #07-01, Matrix, Singapore, 138671 Republic of Singapore; 2 School of Biological Science, Nanyang Technological University, 60 Nanyang Drive, Singapore, 637551 Republic of Singapore; 3 Aklilu Lemma Institute of Pathobiology, Addis Ababa University, Addis Ababa, P.O.BOX 1176, Ethiopia; 4 Detection and Diagnostics Laboratory, Defence Science Organisation National Laboratories, 27 Medical Drive, Singapore, 117510 Republic of Singapore; 5 LKC School of Medicine, Nanyang Technological University, 50 Nanyang Ave, Singapore, 639798 Republic of Singapore; 6 National Public Health Laboratory, Ministry of Health, Singapore, Republic of Singapore; 7 Department of Biological Sciences, National University of Singapore, 8 Medical Drive, Singapore, 117597 Republic of Singapore; 8 Current address Genome Institute of Singapore, A*STAR, 60 Biopolis Street, #02-01, Genome, Singapore, 138672 Republic of Singapore

**Keywords:** Macrophages, Avian influenza virus, H5N2, H5N3, Inflammatory response

## Abstract

**Background:**

Influenza A virus (IAV) is a major public health concern, ﻿being ﻿responsible for the death of approximately half a million people each year. Zoonotic transmissions of the virus from swine and avian origin have occurred in the past, and can potentially lead to the em﻿gergence of new IAV stains﻿ in fut﻿ure pandemics. Pulmonary macrophages have been implicated in disease severity in the lower airway, and understanding the host response ﻿of macrophages infected with avian influenza viruses should provide new therapeutic strategies.

**Results:**

We used a systems-based approach to investigate the transcriptome response of primary murine lung macrophages (PMФ) infected with the mouse-adapted H1N1/WSN virus and low pathogenic avian influenza (LPAI) viruses H5N2 and H5N3. The results showed that the LPAI viruses H5N2 and H5N3 can infect PMФ with similar efficiency to the H1N1/WSN virus. While all viruses induced antiviral responses, the H5N3 virus infection resulted in higher expression levels of cytokines and chemokines associated with inflammatory responses.

**Conclusions:**

The LPAI H5N2 and H5N3 viruses are able to infect murine lung macrophages. However, the H5N3 virus was associated with increased expression of pro﻿-inflammatory mediators. A﻿lthough﻿ the H5N3 virus it is capable of inducing high levels of cytokines that are associated with inflammation﻿, this property is disti﻿nct from its inability to﻿ efficiently replicate in a mammalian host.

**Electronic supplementary material:**

The online version of this article (doi:10.1186/s12864-017-3803-6) contains supplementary material, which is available to authorized users.

## Background

Influenza A virus (IAV) represents one of the most important biological threats to human health and the global economy [1–3]. It causes yearly seasonal epidemics and periodic pandemics [4]. Seasonal influenza is estimated to be the cause up to 5 million severe cases and approximately 500,000 deaths each year. There have been four previous influenza virus pandemics, which have been collectively responsible for the deaths of more than 50 million people [5–7]. The combination of drug resistance, adverse side effects and financial costs of drug and vaccine development are significant challenges in the prevention of the disease due to newly emerging influenza virus strains [8, 9].

IAV normally circulates in wild birds, but it can also infect domestic poultry, pigs, cats, dogs, horses, bats and humans. Although direct zoonotic transmission to humans is not common, interspecies transmission from swine and avian to humans can occurr, and zoonotic transmission can contribute to the emergence of new pandemic viruses [10–12]. Based on disease severity in birds, avian IAV can be classified as either being low pathogenic avian influenza (LPAI) (exhibit mild disease symptoms) or highly pathogenic avian influenza (HPAI) (exhibit severe disease symptoms). Influenza A subtypes H5N1, H7N7, H7N3 have been mostly classified as HPAI, while H5N2, H5N3, H9N2, H7N2, H7N9 and H7N10 are commonly identified as LPAI. However, both the LPAI and HPAI can infect humans with varying degrees of disease severity [13, 14]. The morbidities and mortality from HPAI H5N1 since 2003 [15] and LPAI H7N9 since 2013 [16] are examples of severe human disease caused by avian influenza viruses.

Although the mechanisms underlying the transformation of LPAI to HPAI is not always clear, evidence suggests that this is a multifactorial process involving the acquisition of specific sequence motifs in several virus proteins e.g. the polybasic cleavage site in the hemagglutinin (﻿HA) protein [17]. However, there are several instances where the conversion of LPAI to HPAI is not associated with the acquisition of these classical sequence motifs [18], and the mechanisms that lead to transformation into HPAI are poorly understood. Increased pro-inflammatory cytokine expression in the lower airway during influenza virus infection is an important correlate of virus pathogenicity however the mechanism by which specific virus strains induce high levels of pro-inflammatory cytokines expression is not well understood. Since avian species are an important reservoir of influenza viruses, increased understanding of the biological properties of LPAI that are circulating in the natural environment is a prerequisite to understand the risk that they pose in both human and animal disease.

The murine model of influenza virus infection is commonly used to study the transmission, host adaptation, immune responses and pathogenesis of IAVs (reviewed in [19]). However, many human and avian IAVs are not intrinsically pathogenic in mice; hence the virus has to be adapted to the murine host to allow efficient virus replication [19]. Although the mode of species adaption is not fully understood, the available evidence suggests that it involves several factors (e.g. optimization of receptor binding specificity of the hemagglutinin (HA) protein and adaptation of the virus polymerase complex) to allow replication in the cells lining the upper and lower airway [20–22]). Therefore, adaptation of LPAI viruses to a new host involves the introduction of new genetic changes that may mask inherent biological properties of these viruses [23]. The initial sites for IAV infection are the epithelial cells that line the upper respiratory tract (URT), but disease severity is associated with replication in the lower respiratory tract (LRT), and this involves other cell types including the lung resident macrophages [24]. Therefore, the capacity of influenza viruses to cause disease in the animal models depends on their capacity to replicate in the airway cells lining the URT prior to LRT. This biological property is distinct from the capacity of viruses to induce pro-inflammatory cytokines. The analysis of LPAI infection in immune cells isolated from the lung tissue allows us to examine the biological properties of the avian viruses without the prerequisite for productive infection in the upper airway of the mouse.

Macrophages play an important role in disease progression following influenza virus infection, and the host response in macrophage cells following virus infection are important determinants of disease severity. The biological properties of different macrophages are dependent on their tissue location, and macrophages that are resident in the lung exhibit subtle differences in their biological properties when compared to the macrophages resident in other tissues. At least three categories of lung-resident macrophage cells have been described, and are referred to as alveolar, interstitial and exudate macrophages. Additionally, macrophages can be changed from quiescent to activated states in response to external cues, which include cytokines (e.g. *IFNγ*, *IL-4*, *IL-13*, *TGFβ*), microbes and their products (e.g. LPS), growth factors (e.g. *GSF-1* and *GM-CSF*) and other modulators (e.g. phagocytosis following bacterial and viral infections) [24]. Depending on host homeostasis signals and external environment challenges, macrophages present heterogeneous functions such as the release of reactive toxic species, production of pro/anti-inflammatory cytokines and lipid mediators, tissue remodeling clearance of apoptotic cells and oxidized lipoproteins, wound healing and regeneration [24–26]. The interaction between different IAVs and macrophages varies, and the host response to infection is largely virus isolate-specific [27–29]. In this context, there are marked differences in the ability of different virus strains to induce macrophage-related inflammatory mediators, but the mechanisms responsible for this variation are largely unknown. Understanding the molecular mechanisms by which different IAV can induce pro-inflammatory cytokines in virus-infected macrophages may allow the development of therapeutic strategies that can mitigate IAV infection. In addition, this may lead to a better understanding of how viruses that are circulating in the natural environment attain these biological properties, and if they can transfer these properties to other circulating viruses (e.g. by gene reassortment).

We have previously described the biological properties of several LPAI viruses that were isolated from imported live poultry during routine surveillance, which included H5N2, H5N3 and H9N2 viruses [30, 31]. We had concluded that these viruses were representative of LPAI viruses that are circulating in ducks within the SE Asia region. In this current study, we have extended our previous findings on these LPAI viruses and have applied a system-based approach to investigate the host response to these viruses in murine lung macrophages.

## Results

### Primary murine lung macrophages (PMФ) are infected with all the three IAV examined

**Fig. 1:**
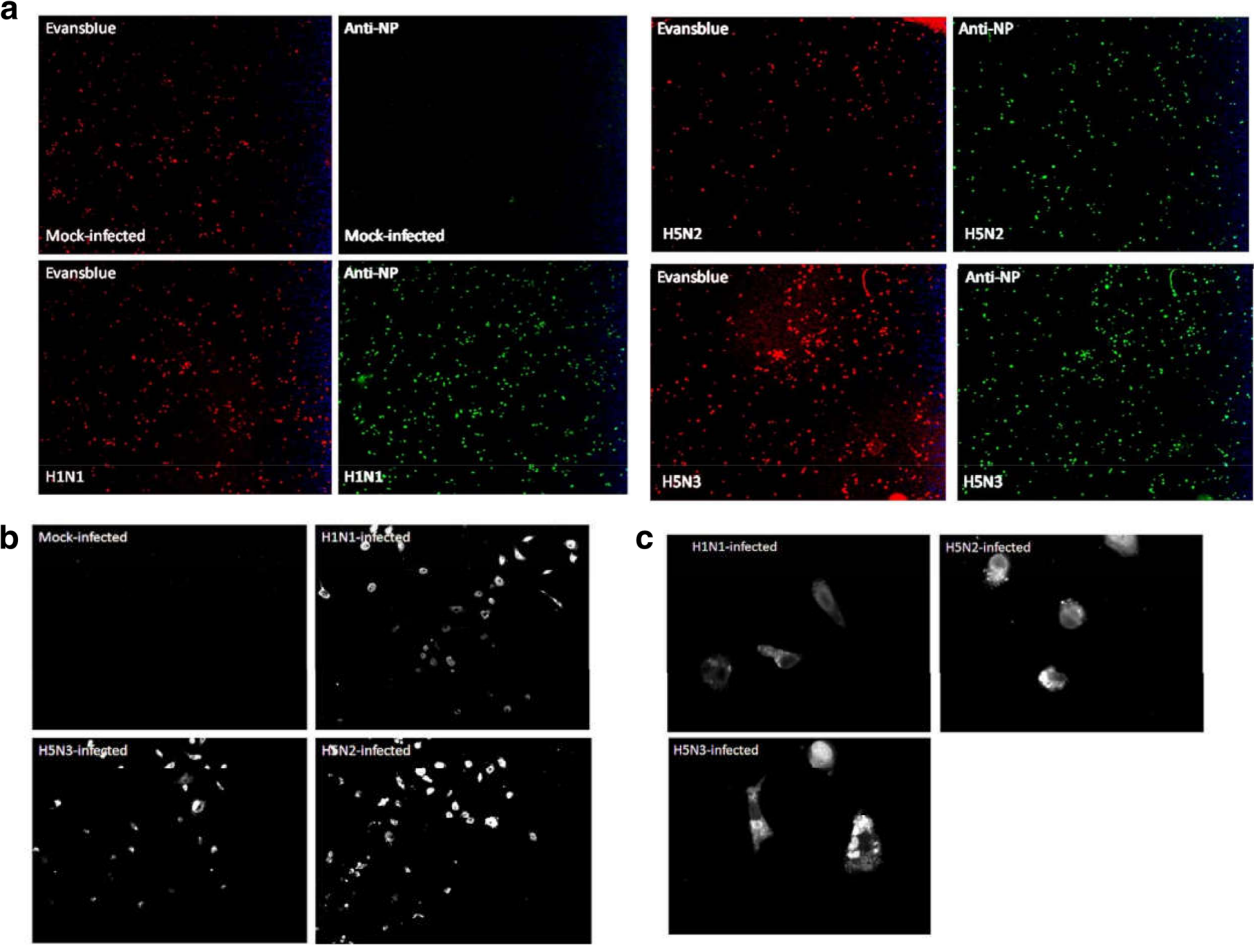
Mouse pulmonary macrophages (PMФ) infected with the H1N1/WSN, H5N2 and H5N3 viruses at 24 hours ﻿post-infection. ﻿**a**) Immunofluorescent (IF) microscopic examination of IAV infected cells (4X objective): Evans blue staining (red colour) indicates the presence of mock and infected cells and anti-NP staining (green colour) shows the presence of virus-infected cells. IF microscopic examination of anti-NP stained mock and IAV infected cells at **b**) 10X microscopic objective and **c**) 40X microscopic objective

We have previously demonstrated that murine alveolar macrophages (AMФ) and PMФ that were isolated from BALB/c mice exhibited similar cytokine induction patterns during respiratory syncytial virus (RSV) infection [32]. Due to logistical issues in obtaining sufficient numbers of AMФ cells from the mice lungs for the microarray analysis, in this analysis, PMФ cells isolated from lung tissue were used. The PMФ were isolated from mouse whole lung tissue as described previously [32], and FACS analysis of anti-CD11b and anti-F4/80 stained PMФ cell preparation cells showed that greater than 95% of the cell population were CD11b+/F4/80+ (Additional file 1: Figure S1A, B). The cells were either mock-infected or infected with each virus using a multiplicity of infection (MOI) of 5. After 24 hours post-infection (hpi) the cells were stained using anti-NP (detects the IAV nucleoprotein (NP)), and visualized using immunofluorescence microscopy (Fig. 1a, b, c). This indicated that in each infected cell assay > 95% of PMФ showed anti-NP staining, and confirmed similar levels of infection with each of the three viruses.

### Global changes in host gene expressions at 24 hours post-infection 

**Fig. 2:**
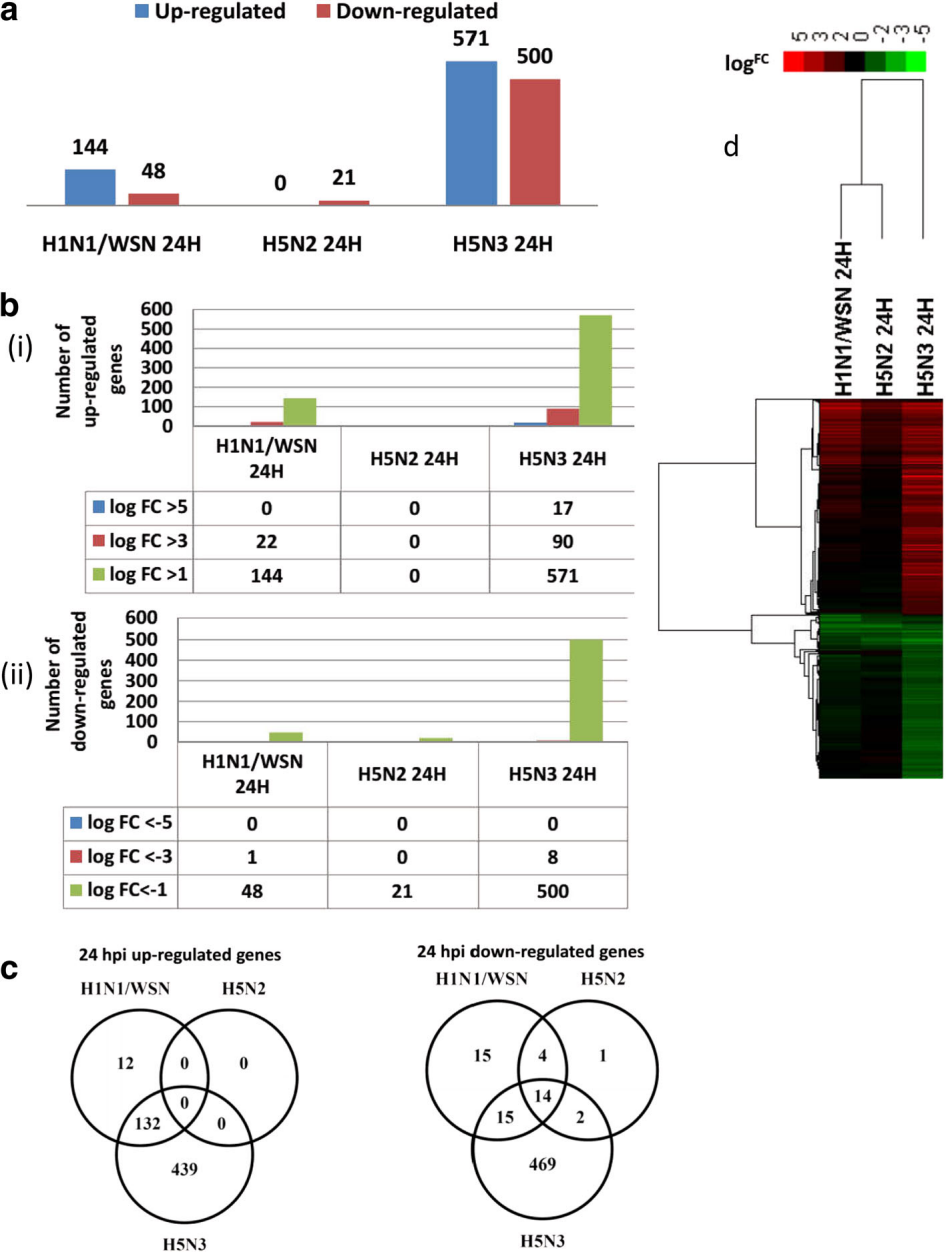
Global host gene expression in mouse PMФ infected with IAVs at 24 hours-post infection (24H)﻿: **a**) Number of up-and down-regulated genes **b**) the extent of up-and down-regulated genes with log^FC^ values i) up-regulated genes ii) down-regulated genes **c**) Overlap analysis of DEGs in three IAV infections **d**) unsupervised hierarchical clustering of DEGs

In the Affymetrix microarray system (GeneChip Mouse Genome 430 2.0 Array) there are 28,974 annotated genes, and analysis of the microarray data indicated that 89.8% (25,886), 89.8% (25,886), 89.7% (25,850) and 84.6% (24,368) of the annotated genes were expressed in H1N1/WSN﻿, mock-infected cells, H5N2 and H5N3 virus-﻿infected cells respectively. The global gene expression in PMФ infected with each of the three viruses was examined at 24 hpi. Overall, 1830 probe sets (1103 genes) were differentially expressed in at least one of the virus infections at 24 hpi (Additional file 2). The numbers of differentially expressed genes (DEGs) at 24 hpi in H1N1/WSN, H5N2, and H5N3 virus-infected cells were 193, 21 and 1071 respectively (Fig. 2a). The numbers of up- and down-regulated genes are 3.9 and 10.4 times higher in H5N3 virus-infected cells than H1N1/WSN virus-infected cells. There are no significantly up-regulated genes in cells infected with the H5N2 virus, and the down-regulated genes in cells infected with the H5N3 virus were 23.8 times higher than in H5N2 virus-infected cells. The number of DEGs with |log^FC^| value > 3 are also higher following H5N3 virus infection (Fig. 2b (i and ii)).

Overlap analysis of DEGs at 24 hpi is shown in Fig. 2c. One hundred thirty-two (91.6%) (132/144) of the genes ﻿showing up-regulated expression in cells infected﻿ with the ﻿H1N1/WSN virus were shared with the H5N3 virus-infected cells. The up-regulated shared genes include *APOL9A*, *CCL12*, *CMPK2*, *DHX58*, *H28*, *IFI44*, *IFIT1*, *IFIT2*, *IFIT3*, *ISG15*, *MX1*, *OAS1A*, *OASL2*, *RSAD2*, *RTP4*, *SLFN3*, *USP18*, *XAF1*, and *ZBP1*. These genes are associated with innate immune response to viral infection and were up-regulated in different IAV strains (H1N1, H3N2, H5N1, H5N2, and H7N1) in A549 cells [33], human blood, PBMC, and primary epithelial cells [34]. These genes (e.g. *IFI44*, *IFIT1*, *OAS1A*, *OASL2*, and *XAF1*) were also implicated in response to other respiratory viruses (RSV and HRV) in human PBMC and lung epithelial cells [33].

Similarly, most (76.2% of the H5N2 virus and 60.4% of the H1N1/WSN virus) down-regulated genes were shared with the H5N3 virus  down-regulated genes. Fourteen down-regulated genes were shared between the three viruses, among which *AGER*, *SFTPD, EGFL6, NDNF*, *DSP* and *TTN* were involved in regulation of cell adhesion and wound healing (Fig. 2c). Approximately 76.8% (439/571) up-regulated and 93.8% (469/500) down- regulated genes were unique to H5N3 infection (Fig. 2c). The hierarchical clustering of DEGs/probe sets at 24 hpi for the three viruses was illustrated in Fig. 2d. In H5N3 virus-infected cells there are unique up-regulated﻿ and down-regulated gene cluster patterns, which wasconsistent with the higher proportion of unique up-and down-regulated genes shown in Fig. 2c. Collectively, the data showed that LPAI viruses induce few common gene expression signatures compared to other IAV isolates and other respiratory viruses, suggesting that most of the DEGs were strain-specific expression signatures.

### Functional annotation of DEGs

**Fig. 3:**
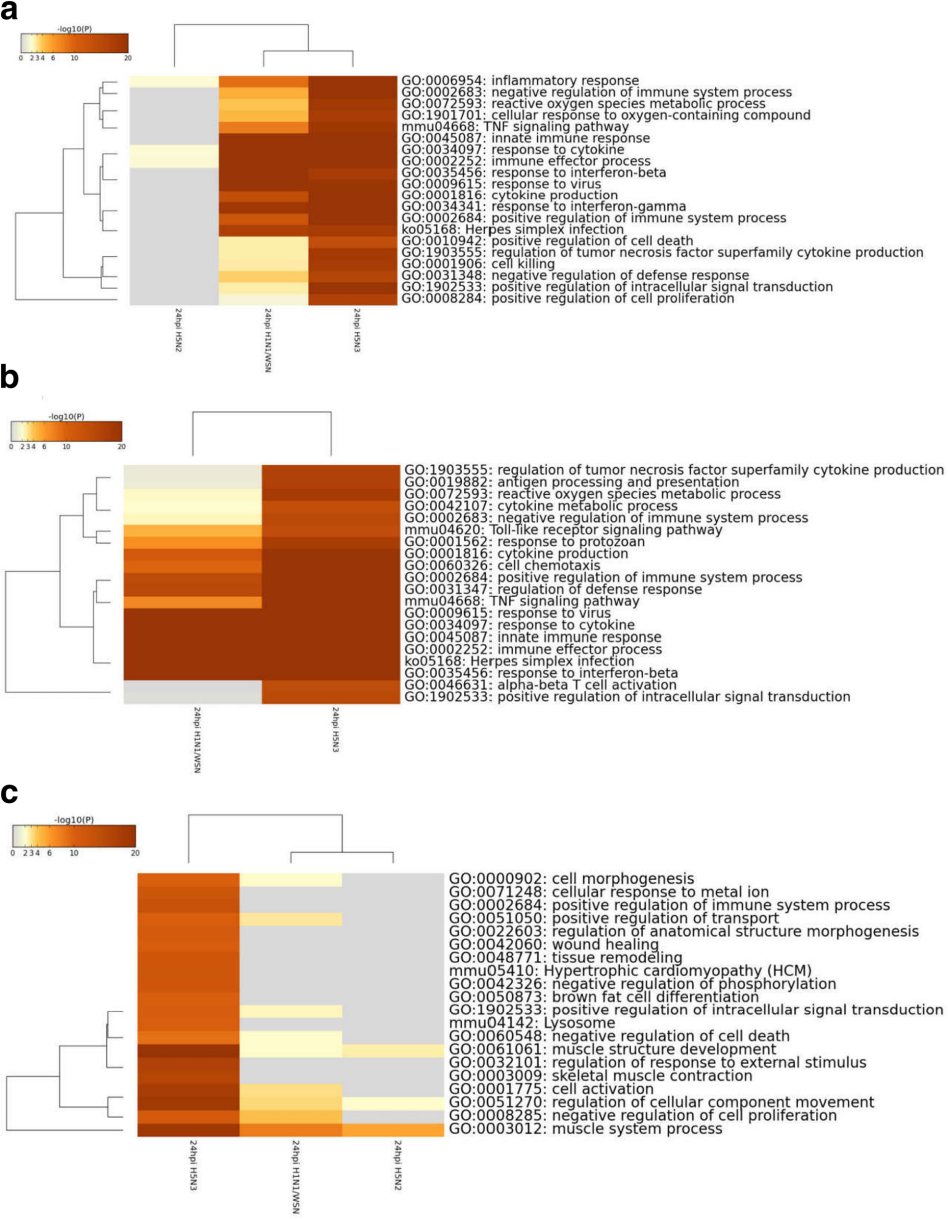
Top 20 statistically significant (q < 0.05) functionally enriched pathways or/and GO biological process (GBP) of the DEGs at 24 hours post-infection (hpi).: **a**) Pathways/GBP from both up- and down-regulated genes. **b**) Pathways/GBP from up-regulated genes **c**) Pathways/GBP from down-regulated genes. Increased intensity of the colour shows increased significance of the pathway/GBP corresponding to each virus infection conditions. More related pathways/GBP were clustered together (left) and the viruses inducing similar pathways/GBP are clustered together (top)

Functional annotations of the DEGs i.e. GO biological process and KEGG pathway was analyzed using Metascape (http://metascape.org/gp/index.html#/main/step1) [35]. We compared significantly enriched pathways and their enrichment p-values (q-value) for all the viruses at 24 hpi. The top 20 statistically significant pathways (q-value < 0.05 ) are described in Fig. 3a. Generally, the pathways are related to immune responses (cytokines, interferons and inflammatory responses), morphogenesis and cell migration (Fig. 3a). Separate pathway enrichment analysis for the up-regulated genes (Fig. 3b) and down-regulated genes (Fig.3c) indicated that the cytokine, interferon and inflammatory responses were enriched in the up-regulated genes, while the enrichment of down-regulated genes were associated with cell migration, morphogenesis and metabolic pathways. It was expected that infection of macrophage cells with IAVs induces immunological responses [36]. The activation of type I and II interferon pathways (Fig. 3a and b) alleviate IAV infections [37], while increased activation of cytokine responses could result in the induction of a cytokine storm (inflammatory cytokines), which are associated with immune-pathological conditions [36, 37]. The H1N1/WSN and H5N3 viruses significantly induced interferon pathways (Fig. 3a and b) and while these viruses induced inflammatory responses, these were more prominent in H5N3 virus-infected cells at 24 hpi. Increased induction of inflammatory cytokines is a characteristic of infections caused by ﻿HPAI viruses, and the induction of inflammatory pathways in H5N3 virus-infected cells could indicate its potential to induce similar pathologic-related immune conditions. Inflammatory cytokines could also disturb the normal cellular structure, morphology and migration contributing to infiltration of fluids and immune cells at the site of inflammation [38]. H5N2 virus infection﻿ showed no induction of inflammatory pathways, deregulation of pathways related to cellular growth, and pathways ﻿related﻿ to﻿ morphogenesis compared with the mouse-adapted H1N1/WSN virus at 24 hpi (Fig. 3b, c). Taken together, this data indicates that the H5N2 virus is less able to induce pathways related to inflammatory cytokines compared to the H1N1/WSN virus, while H5N3 virus induced inflammatory pathways at higher levels.

### Host response to the IAVs infection

Based on the functional annotation and the extent of gene expression changes (highest log^FC^ values), we focused on genes related to cytokines, interferon and cell death and macrophage activation pathways in more detail since these have been implicated in increased virus pathogenicity. Selected genes from virus-induced expression changes were also validated by biochemical cytokine assay.

### IAV induced cytokines

**Fig. 4:**
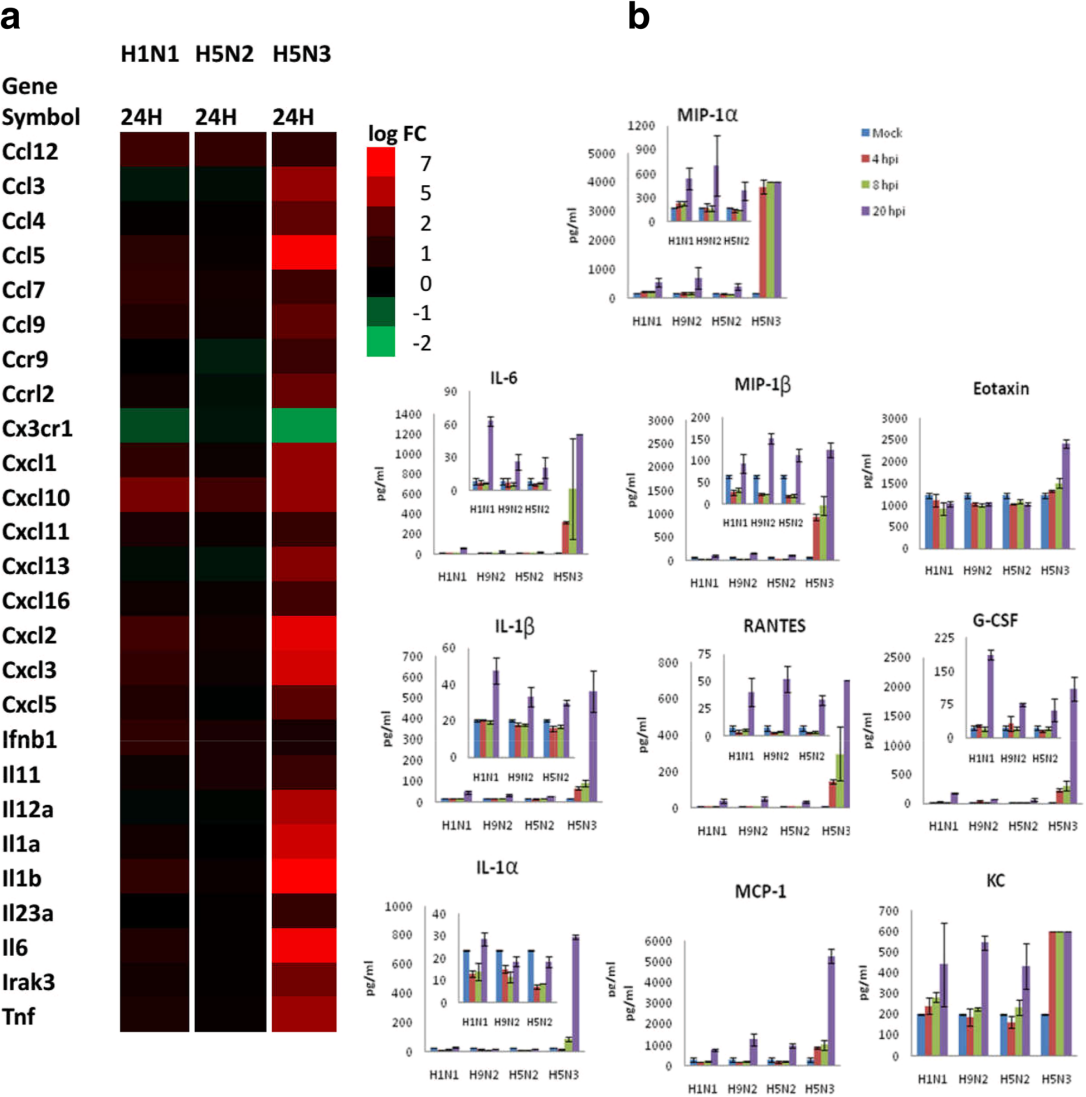
Cytokine and chemokine responses of PMФ in IAV infection **a**) Differentially expressed cytokine and chemokine genes and their expression levels at 24 hours post-infection (24H). **b**) The levels of cytokines and chemokines protein levels measured by biochemical assay at various hours-post infection (hpi).

Infections with the H1N1/WSN and H5N3 viruses induce expression of several cytokine genes in PMФ (Fig. 4a). As indicated in Fig. 4a, the extent of cytokine gene expression following H5N3 virus infection is higher than in cells infected with the H1N1/WSN and H5N2 viruses. To validate the gene expression changes; we examined the cytokine and chemokine response of PMФ in H1N1/WSN, H5N2 and H5N3 virus-infected cells by measuring the cytokine protein levels at 4, 8 and 20 hpi. In addition, we examined another H9N2 LPAI virus that was also isolated from poultry imported into Singapore, and whose biological properties have been examined in cell lines [39]. We observed increased level of inflammatory mediators (*IL-1α*, *IL-1β*, *IL-6*, *MIP-1α (CCL3)*, *MIP-1β (CCL4)*, *RANTES (CCL5)*, *MCP-1 (CCL2)*) following H5N3 virus infection at 4, 8 and 20 hpi compared to cells infected with the H1N1/WSN, H5N2 and H9N2 viruses (Fig. 4b). The cytokine levels measured was consistent with the cytokine gene expression profile, which also provides validation of the gene expression analysis. In cells infected with the ﻿H5N3 virus the levels of cytokine gene expression was significantly higher than in cells infected with either the H1N1/WSN or H5N2 viruses (Additional file 2). At the protein level, the cytokines in H5N3 virus infection are also 2.3 to 27 times, 2.3 to 48 times and 3.3 to 40 times higher compared to H1N1/WSN, H5N2 and H9N2 virus infection respectively (Fig. 4b). In contrast, the expression of cytokines that are important for the survival of macrophages was down-regulated in H1N1/WSN and H5N3 virus-infected cells at 24 hpi (Fig. 4a) [40]. Taken together, inflammatory chemokines and cytokines are highly induced in H5N3 virus-infected cells, compared with cells infected with the H1N1/WSN, H5N2 and H9N2 viruses.

### Interferon stimulated genes (ISGs) and cell death

**Fig. 5:**
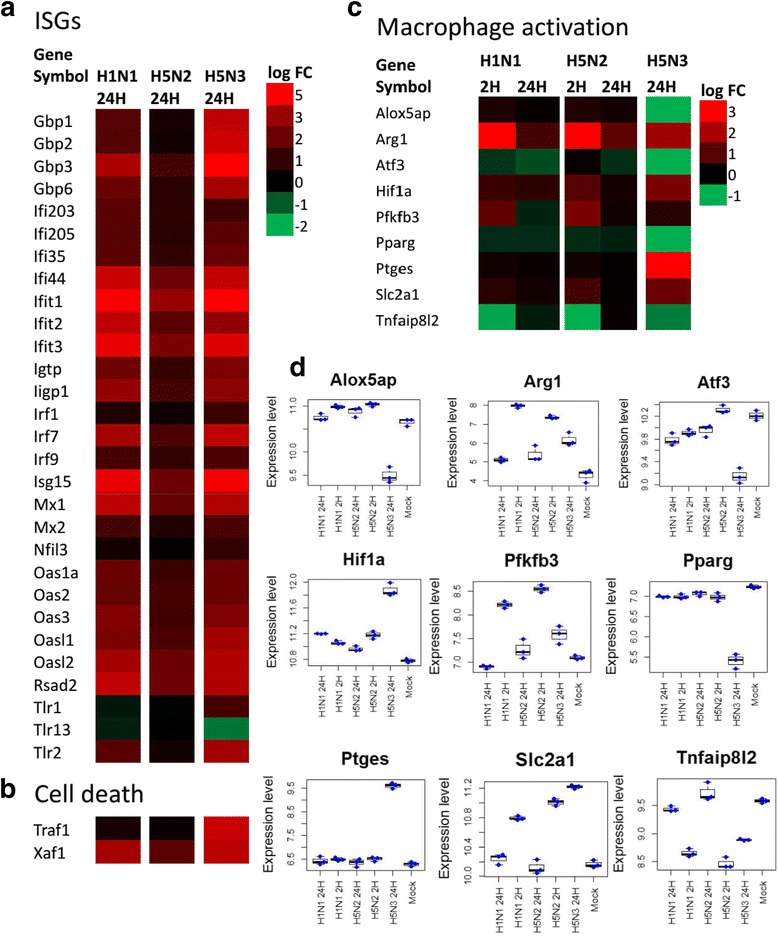
The expression levels of PMФ genes associated with **a**) ISGs, **b**) cell death, **c**﻿) macrophage activation. **d**) Boxplot and bess warm plots indicating the expression distribution of the DEGs involved in macrophage activation. Date obtained at 2 (2H) and 24 (24H) hours-post infection is indicated.﻿

Virus infections are first recognized by the pattern recognition receptors of the host cell, and subsequently induce activation of IRFs and production of interferon (*IFN*). This can be followed by transcription of many antiviral ISGs [41]. The ISGs have several cellular effects, which include anti-viral, immuno-modulation, anti-angiogenic, cell cycle arrest and anti-apoptotic functions [42]. As expected, several of the ISGs were induced in PMФ upon infection with the H1N1/WSN and H5N3 viruses (Fig. 5a). Unlike the higher expression of cytokine responses in H5N3 virus infection, generally the extent of ISGs expression were similar in H1N1/WSN and H5N3 virus infection (Fig. 5a). However, certain ISGs (e.g. guanylate binding protein 1, 2, 3 and 6 (*GBP1*, 2, 3 and 6)) have higher expression levels following infection with the H5N3 virus than the other two isolates (Fig. 5a). *GBP1*, *GBP2* and *GBP3* have antiviral activity against IAV [43] and can also be activated by inflammatory cytokines [44]. *IFIT1* is significantly expressed in both H1N1/WSN and H5N3 virus-infected cells. It is an antiviral protein for viruses that bear 5′-triphosphate RNA [45], however recently it was reported to have no effect on negative sense RNA viruses including influenza [46]. Raw264.7 cells pre-treated with *TNFα* presented with an elevated level of *IFIT1* induced cell death [47]. In line with this, in H1N1/WSN and H5N3 virus infection an elevated level of *IFIT1* was associated with increased expression of anti-apoptotic genes XIAP associated factor 1 (*XAF1*) in both viruses and TNF receptor-associated factor 1 (*TRAF1*) in H5N3 virus infection  (Fig. 5a) in response to early cell death [48].

### Macrophage activation

There is growing evidence that macrophages are functionally subtyped into M1 (activated by bacterial lipopolysaccharides (LPS) or IFNγ) and M2 (activated by IL-4, IL-13 or TGFβ). M1 macrophages favour glycolytic metabolism, production of reactive oxygen species (ROS) and pro-inflammatory reactions, and are associated with a Th1 response which is essential to combat intracellular infections. The M2 macrophages utilize fatty acid oxidation and mitochondrial respiration and induce anti-inflammatory mediators and are linked to Th-2 response, which is important for extracellular parasitic responses, tissue remodeling, and repair and wound healing. M1 and M2 have a different metabolic profile ([24–26, 49–53]). M1 cells are characterized by overexpression of *NOS2*, *MTOR*, *PFKFB3*, *COX2*, *PTGES*, *HIF1A*, *SLC2A1*, *G6PD*, *GCK* and down-regulation of *DHCR24*, *COX1*, *LTA4H*, *TBXAS1*, *ALOX5*, and *LCAT*. On the other hand, M2 cells showed increased expression of *ARG1*, *PRKAB1*, *PFKFB1*, *STAT6*, *COX1*, *ATF3*, *PPARA*, *PPARG*, *SDH*, *NRF1*, *ESRRA*, *TNFAIP812*, *EPAS1*and low level of *PTGES*.

We examined if the expression changes in IAV infected PMФ lead to the formation of either of these macrophage phenotypes. The expression changes of M1/M2 associated metabolic genes indicated that H5N3 virus induces classical activation of PMФ presenting with M1 like phenotype with up-regulation of *SLC2A1* and down-regulation of *PPARG*, *ATF3* and *TNFAIP812* (Fig. 5c, d). Similarly, genes associated with wound healing and tissue remodeling were down-regulated in cells following H5N3 infection (Fig. 3c). In contrast, H1N1/WSN and H5N2 virus infection induced a M2 like phenotype, which is characterized by increased expression of arginase 1 (*ARG1*) at 2hpi (Fig. 5c, d) and had a similar metabolic profile as unpolarized (inactivated) macrophages (mock infected) (Figs. 3c, 5c, d) [54]. Taken together, this data suggests that while H1N1/WSN and H5N2 viruses induced M2 like activation, the H5N3 virus induces M1 phenotype macrophages.

## Discussion

Influenza virus infection of alveolar epithelial cells type II (AECII) and murine lung tissue showed increased expression of cytokines (*IL-6*, *CXCL10*, *CXCL5*, and *CXCL9*) and ISGs (*RSAD2*, *IFIT1*, *IFI44*, *IFIT2*, *MX1*, *OASL2*, *GBP3*, *IIGP1*, *SLNF4*). Although the levels of expression differed in these studies, several of the inflammatory cytokines and interferon response were detected in both the lung and AECII transcriptome analysis [55]. Similarly, several other cell types of the lung e.g. epithelial cells type I, macrophages, dendritic cells, and mast cells contribute to inflammatory cytokines production during IAV infections [56–58]. We observed similar increases in expression of inflammatory cytokines in the H5N3 virus-infected PMФ cells.

The H5N3 virus induced higher levels of ﻿pro-inflammatory cytokine levels in mouse PMФ compared with the H1N1/WSN and H5N2 viruses. Increased inflammatory cytokine expression in whole mouse lung tissue infected with different mouse-adapted pathogenic/virulent IAV subtypes have been described previously using a transcriptomic approach [55, 59–65]. The 2009 pandemic H1N1 virus induced increased expression of *CXCL10*, *OASL*, *OAS2*, *IFIH1*, *USP18* and *XAF1* [65]. Similarly, up-regulated expression of inflammatory cytokines (*CXCL10*, *CXCL11*, *CXCL9*, *CXCL13*, *CCL9*, *CCL5*, *IL-6*, *IL1*, *IL1A*, *IL1B*, *TNF*, and *TNF-α*) and ISGs (*GBP2*, *GBP4*, *GTP1*, *ISG15*, *DdX58*, *IFIH1*) were shown in virulent H3N2/HK31 virus infections [59–61]. In addition, the lung transcriptome of HPAI H5N1 virus infection in ﻿﻿susceptible mouse revealed increased expression of inflammatory cytokines (*CCL2*, *CXCL2*, and *TNF-α*, *CSF3*) and interferon responses (*IFN-α*, *IFN-β*, *IL28B*, *IFNA4*, *IFNA5*), which were associated with increased viral load and disease severity [62, 63]. The H7N9, H7N7 and H5N1 infected mouse lung transcriptome also showed higher levels of *CXCL13, IL-6*, *IFNG*, *IFNB1*, *IRF9*, *IRF1*, and *TNF* compared with 2009 pandemic H1N1 virus infections. Furthermore, increased expressions of inflammatory cytokines and decreased transcription of genes associated with lipid metabolism correlated with increased disease severity [65].

Lung-resident macrophages have been implicated in disease severity following IAV infection [27, 66]. The pathogenicity of IAVs correlated with their ability to induce high levels of pro-inflammatory cytokines in macrophages [67], which in turn is proposed to cause the increased migration of granulocytes into the lung that leads to localized tissue damage [68]. Although the association of high levels of infectious virus production and higher levels of inflammatory responses is suggested, other factors may also play a role in this process. The PR8/H1N1 virus strain is highly virulent and was characterized by higher level of virus production and lung inflammation than HKx31/H3N2 and BJx109/H3N2 viruses [69]. Characterization of inflammatory cell types in mice lung showed that overexpression of *CCL2* and *CCR2*+ monocyte-derived cells (monocyte-derived dendritic cells and exudate macrophages) are the predominant cause of immune pathology during PR8/H1N1 virus infection [70]. In addition, TRAIL-expressing macrophages (exudate macrophages) are implicated in influenza-induced pneumonia through increased expression/release of *TRAIL* and alveolar epithelial cell (AEC) apoptosis [71]. The HPAI H5N1 virus is a potent inducer of of inflammatory mediators (e.g. *IL1*, *IL-6*, *TNFα*, *IL-8*, *MCP-1*, *IP-10*, *IFNγ*) in patients, *in vivo* mouse model experiments and *in vitro* macrophage infections [67, 72–78].

Although all the three viruses infected PMФ with similar efficiency, surprisingly the H5N3 virus induced highest inflammatory responses at 24hpi compared to H1N1/WSN and H5N2 viruses. However, we obtained no evidence that the infection of murine macrophages with either of the viruses induced a productive infection (Sutejo, Tan and Sugrue, unpublished observations). In this context mouse macrophages infected with either of H1N1/WSN, A/HK/483/97 H5N1 (HPAI) or A/X-31/H3N2 viruses showed induced﻿ expression of inflammatory cytokines through activation of either *NF-KB* or *MAPK* signaling pathways in a strain dependent manner [79–83]. However, it was reported that a productive infection in murine-derived macrophages was characteristic of the HPAI H5N1 virus [79]. Although these studies have indicated stain-specific differences in pro-inflammatory cytokine induction in murine macrophages, it is not clear if these observations can be extrapolated to human macrophages. However, HPAI H5N1 infection lead to the expression of higher levels of inflammatory mediators than seasonal H1N1 and H3N2 viruses in human AM [73, 84]. Moreover, the avian viruses H5N1 and H7N9 induced higher inflammatory responses than the seasonal or 2009 H1N1 pandemic viruses [67, 74, 85–90].

Interestingly, H5N3 virus-infected PMФ cells induce expression patterns and pathways (including cytokines) that are similar to the response that we have observed in RSV-infected PMФ cells (Additional file 1: Figure S1C, D; Additional file 2) [32]. RSV is another important virus that can cause lung tissue damage via pro-inflammatory cytokine induction in the lower airway. Therefore comparing the cellular response to these viruses may give insights into the interaction that these viruses with macrophages, and possibly the induction of common pathways that may be activated during virus infection that leads to inflammation. We compared the DEGs in this study with DEGs of PMФ in response to RSV infection from our previously published work [32]. Overlap analysis of the up-regulated genes in IAVs infection with RSV indicated 465 (277 up-regulated  and 188 down-regulated), 102 (100 up-regulated and 2 down-regulated) genes shared in H5N3 and H1N1/WSN virus infected cells respectively (Additional file 1: Figure S1C), indicating, higher numbers of DEGs shared between RSV and the H5N3 virus than with the H1N1/WSN and H5N2 viruses. The severe form of diseases due to RSV infection is associated with increased level of inflammatory cytokines and chemokines (*TNFα*, *IL-1αβ*, *CXCL8*, *CXCL10*, *CCL5*, *IL6*, *CCL2* and *CCL3*) production by epithelial cells and macrophages (reviewed in [91, 92]). Similarly, RSV-infected PMФ showed an elevated level of these inflammatory mediators [32]. The levels of either gene expression or cytokine proteins (*IL-1α*, *TNFα*, *IL-6*, *CXCL10*, *CCL5*, *CCL2*, *CCL3*, *CCL4*, and *CCL5*) in RSV-infected PMФ are comparable to the H5N3 virus infection (Additional file 2). This suggests that the H5N3 LPAI virus and RSV could share common features in the way that they interact with the macrophages.

Since the murine macrophage cells were equally susceptible to infection with each of the three viruses these differences cannot be explained by reduced infections rates. The H5N3 virus internal genes have been shown to share common ancestry with different subtypes of avian viruses circulating in different geographical locations and environment [31]. This previous analysis revealed a high level of sequence conservation between the H5N3 and H5N2 viruses. However, we can hypothesize that small changes in gene sequences may be sufficient to account for the significant differences in the host responses to infection. Although the H1N1/WSN virus was able to replicate efficiently in BALB/c mice, the H5N2 and H5N3 viruses replicated poorly in the mouse infection model (Yeo, Tan and Sugrue, unpublished observations). This is consistent with the expected low efficiency of infection of these avian viruses in mammalian hosts, including humans, and highlights a limitation in the mouse model when examining the interaction of host response in murine macrophages. The capacity of the H5N3 virus to induce pro-inflammatory cytokines in macrophages is therefore a property of the virus that is distinct from its capacity to replicate in non-avian cell types. We have no evidence that this biological property is unique to the H5N3 virus examined in this study, and it is possible that the capacity to induce high levels of cytokines may also be a property of other circulating LPAI viruses. The expected low replication rates in non-avian hosts may normally mitigate this intrinsic biological property of such viruses. However, we can speculate that in the natural environment such viruses (e.g. the H5N3 virus) could transfer their property of inducing high levels of pro-inflammatory cytokines to another virus that replicates better in mammals. Assuming that such a reassorted virus would be viable in the natural environment, this could result in inherent biological changes with medically important implications. It is therefore, necessary to understand this process more completely,  and identify sequence motifs that may correlate with this biological property (e.g. using the FluSurver platform: http://flusurver.bii.a-star.edu.sg). This information would be of use in surveillance studies which would allow the threat of circulating viruses to be better assessed.

The complete mechanism of avian IAV adaptation and pathogenicity to mammalian hosts is not well understood yet. However, it is likely that it involves the HA ﻿protein receptor binding specificity and polymerase complex activity [93]. In terms of key host specificity markers like HA 226 (Q avian, L mammalian, H3-based numbering) and PB2 627 (E avian, K mammalian), the H5N3 virus strain studied here still has the avian-preferred residues in both cases. Interestingly, the HA does have one S159N mutation that has been implicated in increased binding to mammalian-type 2,6 receptors without loss of binding to avian-like 2,3 [94]. However, the same mutation is also found in the H5N2 virus strain used in this study so it cannot be held responsible for the observed differences. On the other hand, we have found a K615R mutation in the PA protein of the H5N3 virus isolate, but not in the H5N2 and H9N2 viruses used in our study. Combination of mutations in internal genes, including PA 615R, has been suggested to contribute to the virulence of avian viruses in mammalian hosts [95]

Under normal circumstance, a cell has its own inherent metabolic activity that is responsible for a variety of different physiological processes. Increasing evidence suggests that metabolism under the influence of specific stimuli (e.g. virus infection) is an important determinant for functional phenotype differences in macrophages [50]. There are two mechanistic options to connect macrophages and metabolism, i.e. either the macrophage cell can regulate metabolic function extrinsically, or intrinsic metabolic functions dictates macrophage activation states [50]. The current study demonstrated increased expression of *ARG1* in H1N1 and H5N2 virus﻿-infected PMФ at 2hpi. In M2 activated macrophages increased expression of *ARG1* catalyzes polyamine production, which is essential for the synthesis of collagen, tissue remodeling and cell proliferation [51]. In line with this, following  H1N1/WSN and H5N2 virus infection there is a remarkable change in the maintaining of the down-regulated pathways that were associated with cell proliferation and cellular organization from 2hpi to 24hpi (Fig. 3c, Additional file 1: Figure S1E). In contrast, M1 macrophages are characterized by increased expression of NOS2 and NO production. The subsequent induction of *NF-kB* mediated inflammatory mediators (*TNF-α*, *IL-6*, *IL-12*, NO and others) leads to tissue inflammation and cell death. In addition, the M1 macrophages reprogram glucose metabolism to the glycolytic pathway and the pentose phosphate pathway (PPP), which results in the production of reactive oxygen species (ROS) that mediate tissue disruption [51]. Similarly, the current study revealed that the H5N3 virus activated the PMФ into a M1 phenotype which is characterised by increased expression of *NF-kB* mediated inflammatory cytokines and down-regulation expression of pathways related to proliferation and myogenesis. Although the effect of different IAV strains in activation of different macrophages subtypes is not well understood, recently Zhao et al., 2014 suggested that activation of macrophages into M1/M2 phenotype is time and macrophage type dependent [96]. Continuous activation of macrophages into the M1 phenotype is associated with severe disease, while the M2 phenotype is an indication of recovery from disease/infection [96].

This analysis demonstrates that LPAI viruses interact with immune-related cells in a virus-specific manner, leading to virus stain-specific changes in pro-inflammatory cytokine expression. The capacity of the avian H5N3 virus to infect mammalian host cells and result in increased expression of inflammatory mediators suggests that this is a unique property that is distinct from other factors, e.g. virus replication in mammals. Since in the natural environment the H5N3 virus can potentially reassort with other circulating influenza viruses, and if such virus is viable this may be able to transfer unique properties that are associated with increased cytokine induction. The identification of sequence motifs in the H5N3 virus that is associated with the increased cytokine induction will be the subject of future studies. This information will allow the identification of sequence motifs that can be used in surveillance studies to assess LPAI viruses which could impose threats to human health in the future.

## Conclusion

The H5N2 and H5N3 LPAI viruses can infect PMФ similarly to the mouse-adapted H1N1/WSN virus. The global and temporal host gene expression indicated that while there were comparable expression levels of ISG in H1N1/WSN and H5N3 virus-infected cells, increased pro-inflammatory responses were associated with H5N3 virus infection. This systems-based approach provides evidence that increased cytokine induction during influenza virus infection is not a property that is unique to HPAI viruses, but it is also an inherent property of some LPAI viruses. A better understanding of the signals in LPAI viruses that induce host responses to infection should provide insights into the molecular mechanism of pathogenesis. It will also allow a better understanding of the contribution that these viruses play in the emergence of novel influenza strains in epidemics and pandemics.

## Methods

### Virus, tissue culture and antibodies

H1N1/WSN virus (A/WSN/1933(V-1520)) was purchased from American Type Culture Collection (ATCC). The LPAI isolates H5N2/F118 (A/Duck/Malaysia/F118/2004), H5N3 (A/Duck/Singapore-Q/F119/1997) and H9N2 (A/Duck/Malaysia/02/2001) viruses were obtained from Agri-Food and Veterinary authority of Singapore and have been characterized previously [31]. The 9 to 11-day old embryonated chicken eggs were used for virus stock preparation, and TCID50 in MDCK (standard plaque assay) was performed to measure the infectivity of the viruses. PMФ (CD11b+) cells were obtained from 6-8 pathogen-free female BALB/c mice lungs as described before [32]. Briefly, single cell suspensions (0.5% BSA, 2 mM EDTA, in 1xPBS) obtained from digested murine lungs (with collagenase D (1 mg/ml; Gibco) was passed through a 30 μm filter. CD11b microbeads and a LS positive selection column (MiltenyiBiotec) were used to purify CD11b + cells. The purified cells were confirmed by Trypan blue viability assay and more than 95% of them were viable. L929 cell (conditioned 30%v/v) medium was used to maintain the purified cells at 37 °C in 5% CO_2_. The cells were washed with PBS to remove non-adherent cells before used for virus infection. The NP, F4/80-FITC and CD11b antibodies were purchased from Chemicon, Biolegend and MiltenyiBiotec respectively. Cells were infected with each virus using a MOI of 5.

### Immunofluorescence (IF) microscopy

The cells were on 13 mm cover slides and treated with 4%paraformaldehyde in PBS prior to permeabilization using 0.1% triton X100 in PBS. The cells were then stained with primary anti-NP antibody and FITC conjugated anti-mouse secondary antibody (IgG). The stained cells were mounted on slides using Citifluor and examined using Nikon eclipse 80i fluorescent microscope.

### Cytokine protein assay

Supernatant both from mock-infected or virus﻿-infected ﻿PMФ was obtained and centrifuged at 10,000 g for 10 min at 4 °C and the refined supernatant was used for cytokine assay. The cytokines in the supernatant were analyzed according to the Bio-Plex protein array system using the Bio-Plex mouse cytokine 23-plex panel (1x96 –well, #M60009RDPD, Bio-Rad).

### Microarray analysis

PMФ were either mock-infected or virus-infected and at 2 and 24hpi (in H1N1/WSN and H5N2/F118) and 24hpi (H5N3) the cells were harvested at 4 °C using RNA later (Ambion) in PBS buffer, and stored at -80 °C. Three independent experiments were performed for each time point and virus infections. RNeasy mini kit (Qiagen) was used to extract total RNA and double-stranded cDNA was synthesized from 3 μg of total RNA using the GeneChip One-Cycle cDNA synthesis kit (Invitrogen, Affymetrix). Biotin-labelled cRNA was then synthesized using the GeneChip IVT labelling kit (Affymetrix). After cRNA fragmentation, 15 μg of labelled cRNA was hybridized to the GeneChip Mouse Genome 430 2.0Array (Affymetrix). The arrays were scanned with GeneChip scanner3000 (Affymetrix) after they were washed and stained using the GeneChip Fluidics Station 450 (Affymetrix). Affymetrix.CEL files were generated from GeneChip Operating Software (GCOS) and imported to AltAnalyze 2.1.0 for analysis [97]. Proper quality control and normalization were performed in accordance with the AltAnalyze for Affymetrix.CEL files. The mean expression for each biological group and mean detection above background probabilities were calculated for each feature (probe). Expression values were calculated if the mean expression and the mean detection above background probabilities for at least one group meet expression thresholds, expression >70 and *p*-value < 0.05. Differentially expressed genes (DEGs) or probe sets were selected with statistical significant change (P-value of the student's t-test (raw p-value), ANOVA and the Benjamini-Hochberg False Discovery method (adjusted p-value) < 0.05) and |Log Fold change (FC)| > 1 (Changes in virus infection in respect to corresponding mock). In the case where a gene is represented by multiple differentially expressed probe sets, the probe with maximum logFC value was selected to construct heat maps. The number of DEGs was determined after summarizing the gene symbols of the differentially expressed using a customized Perl script (by removing redundancies). Venn diagrams showing the overlap of genes in different groups was performed using Venny 2.1 [98]. Functional annotation and pathway enrichment analysis were performed using Metascape (http://metascape.org/gp/index.html#/main/step1) [35] and q-value < 0.05 was used to select statistically significant pathways. The data discussed in this publication have been deposited in NCBI's Gene Expression Omnibus [99] and are accessible through GEO Series accession number GSE86446

(https://www.ncbi.nlm.nih.gov/geo/query/acc.cgi?token=exkdcksmdhylbcn&acc=GSE86446). Unsupervised hierarchical clustering was used to cluster the DEGs using Gene Cluster version 3 [100].

## Additional files

Additional file 1: Figure S1. **A**) Identification of PMФ using ant-F4/80 antibody and ant-CD11b antibody. **B**) FACS analysis of anti-CD11b and anti-F4/80 stained PMФ. **C**) Overlap analysis of DEGs in three IAV viruses and RSV (the data for RSV was obtained from our previously published work (Ravi et al., 2013)). **D**) Top 20 significantly enriched pathways of DEGs in IAVs and RSV infected PMФ. **E**) Unsupervised hierarchical clustering of DEGs at 2 and 24hpi in H1N1/WSN and H5N2 infections. (PDF 230 kb)

Additional file 2: List of differentially expressed probe sets or genes **A**) List of all DEGs with log^FC^ and adjusted p-values **B**) Expression levels of cytokines in H5N3 compared to H1N1/WSN and H5N2 **C**) Cytokine expression levels in H5N3 and RSV. (XLSX 233 kb)

## Abbreviations

DEGs: Differentially expressed genes
HA: Hemagglutinin
HPAI: Highly pathogenic avian influenza
hpi: Hours post-infection
IAV: Influenza A virus
ISGs: Interferon-stimulated genes
KEGG: Kyoto Encyclopedia of Genes and Genomes
LPAI: Low pathogenic avian influenza
LRT: low respiratory tract
M1: Macrophages activated by bacterial lipopolysaccharides (LPS) or IFNγ
M2: Macrophages activated by IL-4, IL-13 or TGFβ
MOI: Multiplicity of infection
NP: Nucleoprotein of Influenza A virus
PMФ: Primary murine lung macrophages
URT: Upper respiratory tract
