# Supplementary material for: Systems-based approach to examine the cytokine responses in primary mouse lung macrophages infected with low pathogenic avian Influenza virus circulating in South East Asia
Source: BMC Genomics. 2017 May 30;18:420. doi: 10.1186/s12864-017-3803-6 (PMC5450074; doi:10.1186/s12864-017-3803-6)
Supplement: Supplementary file 1 — A) Identification of PMФ using ant-F4/80 antibody and ant-CD11b antibody. B) FACS analysis of anti-CD11b and anti-F4/80 stained PMФ. C) Overlap analysis of DEGs in three IAV viruses and RSV (the data for RSV was obtained from our previously published work (Ravi et al., 2013)). D) Top 20 significantly enriched pathways of DEGs in IAVs and RSV infected PMФ. E) Unsupervised hierarchical clustering of DEGs at 2 and 24hpi in H1N1/WSN and H5N2 infections. (PDF 230 kb) [file 12864_2017_3803_MOESM1_ESM.pdf]

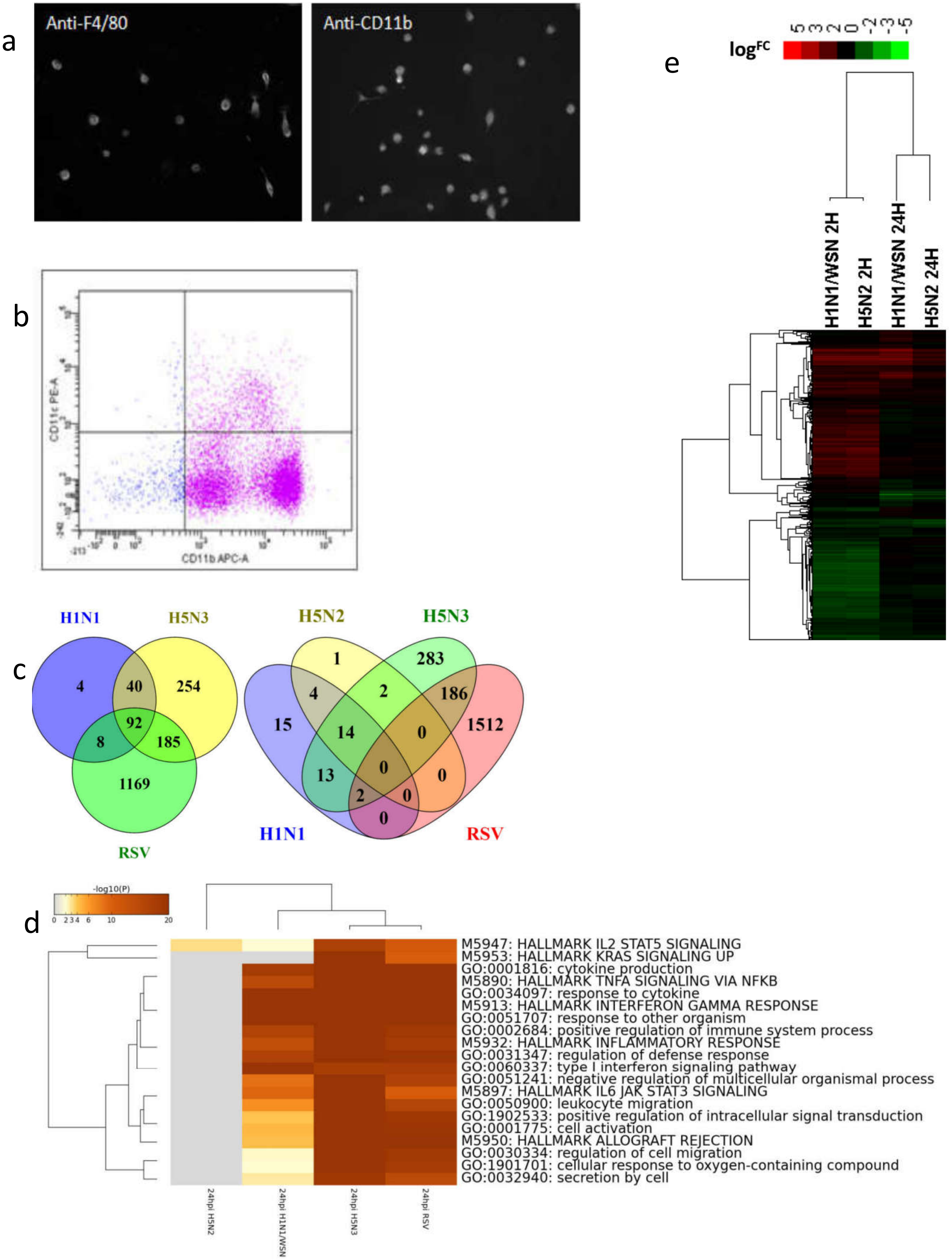
a
Anti-F4/80
Anti-CD11b
b
CD11c PE-A
CD11b APC-A
c
H1N1
H5N3
RSV
4
40
254
92
8
185
1169
H5N2
H5N3
H1N1
RSV
1
283
4
2
186
15
14
0
1512
13
0
0
2
0
0
d
-log10(P)
0 2 3 4 6 10 20
M5947: HALLMARK IL2 STAT5 SIGNALING
M5953: HALLMARK KRAS SIGNALING UP
GO:0001816: cytokine production
M5890: HALLMARK TNFA SIGNALING VIA NFKB
GO:0034097: response to cytokine
M5913: HALLMARK INTERFERON GAMMA RESPONSE
GO:0051707: response to other organism
GO:0002684: positive regulation of immune system process
M5932: HALLMARK INFLAMMATORY RESPONSE
GO:0031347: regulation of defense response
GO:0060337: type I interferon signaling pathway
GO:0051241: negative regulation of multicellular organismal process
M5897: HALLMARK IL6 JAK STAT3 SIGNALING
GO:0050900: leukocyte migration
GO:1902533: positive regulation of intracellular signal transduction
GO:0001775: cell activation
M5950: HALLMARK ALLOGRAFT REJECTION
GO:0030334: regulation of cell migration
GO:1901701: cellular response to oxygen-containing compound
GO:0032940: secretion by cell
24hpi H5N2
24hpi H1N1/WSN
24hpi H5N3
24hpi RSV
e
logFC
5 3 2 0 -2 -3 -5
H1N1/WSN 2H
H5N2 2H
H1N1/WSN 24H
H5N2 24H
